# Accuracy and performance of a new handheld ultrasound machine with wireless system

**DOI:** 10.1038/s41598-019-51160-6

**Published:** 2019-10-10

**Authors:** Enrico Maria Zardi, Edoardo Franceschetti, Chiara Giorgi, Alessio Palumbo, Francesco Franceschi

**Affiliations:** 1 Internistic Ultrasound Service, “Campus Bio-Medico” University, Rome, Italy; 2 Department Upper and Lower Limb Surgery Unit, University Campus Bio-Medico, Rome, Italy; 3 Radiology Department, S. Maria della Misericordia Hospital, Urbino, Italy

**Keywords:** Medical research, Energy science and technology, Health care

## Abstract

We verified the accuracy and performance of a new handheld ultrasound machine, in comparison to a high-end sonographic machine. We performed bilateral measurements of the following tendon districts (supraspinatus, flexor of the middle finger, patellar and Achilles) and of the cross sectional area of the median nerve in 21 patients using a musculoskeletal ultrasound linear scanner of a handheld sonographic machine and a high-end sonographic machine. Two tail T test was used to evaluate whether there were differences in the measurements between the two sonographic machines. Agreement was evaluated by Pearson’s correlation. The mean time requested for the examinations was 18 and 9 minutes for the handheld and high-end sonographic machines, respectively. No significant differences were found between the measurements obtained with the handheld ultrasound machine and those with the high-end sonographic machine (*p* value ranging between 0.31 and 0.97, according to the examined district), whereas, a moderate correlation was found (*r* coefficient ranging between 0.43 and 0.77, according to the examined district). Although the examination with the handheld ultrasound machine took more time, it showed adequate accuracy and performance; this palmar tool might be also useful in operating rooms.

## Introduction

For over ten years, small portable sonographic machines have been becoming an established concept in the emergency medicine departments to expedite the medical work^1^. The addition of the wireless application to sonographic probes permits a quicker ultrasound scan of the patients directly in their rooms especially to obtain vascular access, implant cardiovascular electric devices or measure neuraxial depth: this also facilitates the establishment of an electronic archival system for an immediate doctor consultation from everywhere^2–4^.

To date, wireless probes are proving to be suitable, fast and safe tools able to overcome certain limits of conventional sonographic machines such as the difficulty to assure a full sterile environment and the need for a second worker to assist in image settings during an interventional procedure^5^. Furthermore, ultrasound guidance becomes easier and safer to use, due to the fact that the eyes of the doctor must not turn but may see in the same direction both the needle puncture site and the ultrasound image display^6^.

Recently, a handheld wireless ultrasound machine, able to connect to any iOS or Android device through a secure Wi-Fi, has been built and launched in the medical world^7^. To validate the clinical applicability of this tool for an orthopedic use, we planned to test it in different parts of musculoskeletal system of 21 subjects, comparing these measurements with those obtained with a high-end sonographic machine.

## Results

Twenty-one patients (10 males and 11 females) with a mean age of 50 ± 21 years and a Body mass index of 24.9 ± 3.5, represented the sample of the study.

The statistical comparison of the mean values of tendon thickness and cross sectional area of the median nerve obtained with the handheld sonographic machine compared to those obtained using the high-end sonographic machine, did not show significant differences (Table 1).

**Table 1:** Mean value of measurements of the thickness of 42 supraspinatus, 42 flexor, 42 patellar and 42 Achilles tendons and of the cross sectional area of 42 median nerves obtained with the handheld and high-end sonographic machines and their statistical comparison.

Measurements	Handheld sonographic machine by Clarius	High-end sonographic machine (GE E9)	*p* Value
Supraspinatus tendon thickness in mm	1.42 ± 0.14	1.36 ± 0.14	0.31
Median nerve cross sectional area in cm^2^	0.0792 ± 0.02	0.0794 ± 0.02	0.97
Flexor tendon thickness in mm	3.44 ± 0.31	3.47 ± 0.31	0.63
Proximal Patellar tendon thickness in mm	3.54 ± 0.7	3.58 ± 0.5	0.76
Mid Patellar tendon thickness in mm	3.07 ± 0.7	3.09 ± 0.6	0.88
Distal Patellar tendon thickness in mm	3.65 ± 0.9	3.48 ± 0.7	0.38
Proximal Achilles tendon thickness in mm	2.68 ± 0.7	2.82 ± 0.5	0.34
Mid Achilles tendon thickness in mm	3.83 ± 0.7	3.77 ± 0.6	0.74
DistalAchilles tendon thickness in mm	3.80 ± 0.6	3.78 ± 0.5	0.84

Pearson′s correlation demonstrated a moderate agreement between the measurements of the two sonographic machines (Table 2).

**Table 2:** Pearson’s correlation index between the measurements performed with the palmar sonographic machine and those with the high-end sonographic machine.

Measurements	*r coefficient*		
Supraspinatus tendon thickness	0.43		
Median nerve cross sectional area	0.75		
Flexor tendon thickness	0.62		
**Tendon segment**	**Proximal**	**Mid**	**Distal**
Patellar tendon thickness	0.70	0.77	0.56
Achilles tendon thickness	0.61	0.53	0.75

Mean and maximum times required for the acquisition of all sonographic parameters were 18 and a half minutes and 25 minutes for the handheld sonographic machine, and 9 and a half minutes and 15 minutes for the high-end sonographic machine. Of note, the time spent to perform the examination with the handheld machine also included that required for the wireless connection; this could vary from day to day depending both on the speed and stability of the company network. Finally, the rechargeable batteries allowed a non-continuous use of a few hours.

## Discussion

The aim of this study was to evaluate accuracy and performance of a new handheld sonographic machine with wireless system. In order to improve the evaluation, a comparison was made between the measurements of tendons and median nerve cross sectional area performed with a new handheld sonographic machine with those of a high-end, using the values obtained with this latter as the reference.

**Figure 1:**
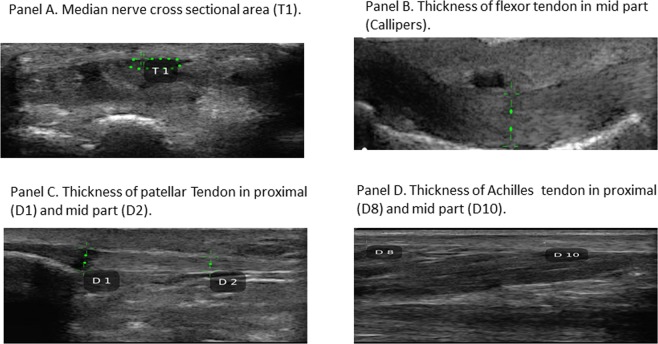
An overview divided in Panels (A–D) of the measurements of the median nerve cross sectional area and of tendon thickness using the handheld sonographic machine.

The quality of images was good and the comparison showed that the handheld sonographic machine is clinically applicable and feasible. The adequate accuracy, reliability and performance ascertained (Fig. 1A–D), encourage the use of this new handheld sonographic machine.

The current period is the era of digitized and miniaturized technology and this palmar tool well meets this requirement. The small size, the not excessive weight and the absence of cables makes it more manageable and easier to use in the operating rooms. Thanks to the presence of more than one battery whose life is of a few hours and rechargeable in just over an hour, any work session may continue without significant interruption. The possibility to wireless upload the images to an electronic archival system, allows to better investigate the images at a later stage, thus facilitating the work.

To our knowledge, this is the first study that demonstrates the suitability for the musculoskeletal use of an handheld sonographic machine with wireless system. A sure limit of this device might be the more time needed to perform the examination than with a high-end sonographic machine, although this was due in part to the speed and stability problems the company network had in those days. Another limit was the lack of the color Doppler software that makes impossible the evaluation of active tendinitis. On the contrary, a notable advantage is the handling and the lower cost than any other sonographic machine. The low cost and the handling of this portable sonographic machine might increase interest among the clinicians in acquiring specific and technical competence in this field. On the other hand, apart from consultations in the medico-legal field, having diagnostic imaging competence may be decisive, as never before, to drive the correct therapeutic decision; therefore, this tool might become useful as a stethoscope for the clinicians.

### Limitations

Since this is a preliminary study, we have to underline the following limitations for a correct interpretation of these data:

- first, the small number of subjects enrolled in the study;
- second, the missing calculation of intraclass coefficient (however, the sonographer that performed the examination is an expert operator that periodically undergoes training programs and constantly controls his performances).

Although a larger cohort of patients should be required to confirm these preliminary results, a moderate correlation was found between the two sonographic machine demonstrating the clinical applicability of the portable tool. This handheld sonographic machine might facilitate the medical activity also in surgery rooms.

## Methods

### Patients

Twenty-one subjects of which 5 sportsmen and 1 sport woman and the other 15 with osteoarthritis, undergoing orthopedic surgery, were consecutively recruited at Orthopedic Unit of our Hospital.

All clinical data [age, sex and body mass index (BMI) that was calculated according to the following formula: kg/m^2^] and the sonographic measurements of the 21 subjects were collected in an electronic database. Ethics approval (46/18OSS) was obtained from the Ethics Committee (Comitato Etico Campus Bio-Medico) of this University.

### Sonographic assessment

A unique operator, experienced in musculoskeletal sonography, conducted the examination basing on international indications and guidelines^8–13^. The regions of interest for this sonographic study were: the shoulder (supraspinatus), the wrist (median nerve), the hand (the flexor tendon of third finger), the knee (patellar tendon), the ankle (Achilles tendon).

Examinations were bilaterally performed in all 21 subjects both with a portable sonographic machine, the palmar “Arthrex Synergy” musculoskeletal (MSK) ultrasound linear scanner “AR-3501B-L7” by “Clarius” “Canada” (multifrequency 4–13 MHz, 167 mm high, 99 mm wide, 42 mm thick and weighing approximately 540 g) and with a high-end sonographic machine, the “General Electric (GE) Logiq E9 XD Clear” “USA”, that adopted a multifrequency (7–16 MHz) linear probe.

After launching a Clarius App for the palmar MSK ultrasound and creating a secure direct WI-Fi connection with iOS or Android mobile devices, the portable sonographic machine becomes almost instantly available to be used and real time images can be transmitted to the selected mobile device. Furthermore, the images can be wirelessly recorded to a secure Cloud site for reviewing and the storage. This palmar tool can be completely immersed in liquid, thanks to a magnesium shell coating and to the absence of cables, thus facilitating any disinfection procedure. A continued use is assured by a rechargeable battery quickly replaceable. A good depth (up to 7 cm) supplied by the scan, allows any kind of orthopedic study.

Preset musculoskeletal examination settings for the best optimization of the images are present in both sonographic machines.

**Figure 2:**
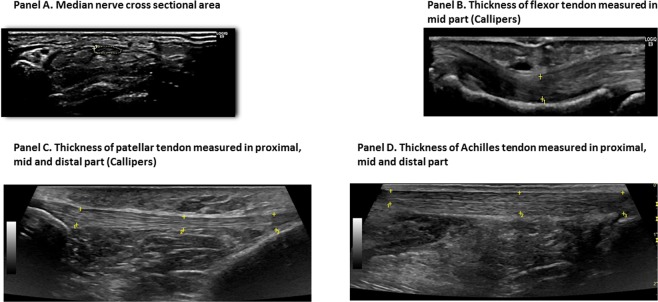
An overview divided in Panels (A–D) of the measurements of the median nerve cross sectional area and of tendon thickness, using the high-end sonographic machine.

The thickness of 42 tendons of each district (supraspinatus, flexor of the third finger, patellar and Achilles) and the cross sectional area of the 42 median nerves were sonographically assessed, in the same manner, with both sonographic machines (Figs 1A–D and 2A–D). Careful attention was placed to define the edges of tendons and of the median nerve, to obtain the best measurements possible of the tendon’s thickness and of the median nerve’s area. During the sonographic examination all patients were at rest and in a relaxed position without passive movements. To reduce the sampling errors linked to the compression of the median nerve and the tendons, the pressure exerted by the probe was minimized. The supraspinatus tendon, the median nerve and the flexor tendon of the third finger were examined with the patient in a seated position. Scanning of the supraspinatus tendon took place when the patient had the palm of the hands on the iliac crest and the elbow directed posteriorly; the maximum thickness of the supraspinatus tendon was measured scanning it in a long-axis view to 1.5 cm from the greater tuberosity of the humerus. Instead, median nerve and flexor tendon of the third finger were scanned with the arms extended on the couch with the palm of the hands upward. The cross sectional area of the median nerve was measured in an axial view, at the proximal inlet of the carpal tunnel at the level at which it was possible to recognize the most palmar point of the pisiform bone, using the direct tracing method with an electronic caliper; the thickness of the flexor tendon was assessed, in a longitudinal view, at the level of the mid part of the proximal phalanx of the third finger.

During patellar tendon examination, the patient was in a recumbent position with the knees slightly bent, whereas the Achilles tendon was scanned with the patient laying prone and the gastrocnemius muscle relaxed.

In the patellar and Achilles tendons the thickness was measured in three points (proximal, mid an distal part) in a longitudinal view. Regarding the patellar tendon, the proximal and distal parts corresponded to the bottom pole of the patella and to the insertion of tibial tuberosity, respectively, while the mid part corresponded to the exact half of the same tendon. Instead, the proximal part of the Achilles tendon corresponded to a point 5 cm away from the calcaneus, the mid part to a point located in the exact half from the proximal and distal parts and the distal part to the point of tendon’s insertion of the calcaneus.

The mean value of all measurements from the right and left side was used for statistical evaluation. The time spent for each sonographic assessment was also taken to evaluate the feasibility.

### Statistical analysis

Data were analysed by graphpad Software SPSS® version 22.0 (IBM Corporation, Chicago, IL, USA). Clinical data (i.e. age, BMI) were expressed as the mean ± SD. Two tail t Test was performed to analyse whether there were differences between the results obtained in any district by the two sonographic machines. Pearson’s rank coefficient was finally used to assess the presence or absence of the correlation between the measurements obtained by the two sonographic machines.

### Ethical approval

All procedures performed in studies involving human participants were in accordance with the ethical standards of the institutional and/or national research committee and with the 1964 Helsinki declaration and its later amendments or comparable ethical standards.

### Informed consent

Informed consent was obtained from each subject. Ethics committee of this University approved the study.
